# Health service utilization among autistic youth in Aotearoa New Zealand: A nationwide cross-sectional study

**DOI:** 10.1177/13623613241298352

**Published:** 2024-12-03

**Authors:** Laurie K McLay, Philip J Schluter, John Williams, Francesca Anns, Ruth Monk, Joanne Dacombe, Gabrielle Hogg, Jessica Tupou, Troy Ruhe, Taylor Scott, Emma Woodford, Hiran Thabrew, Nicholas Bowden

**Affiliations:** 1 Te Whare Wānanga o Waitaha, University of Canterbury, New Zealand; 2 The University of Queensland, Australia; 3 University of Otago, New Zealand; 4 The University of Auckland, New Zealand; 5 Independent Researcher, New Zealand; 6 Te Herenga Waka, Victoria University of Wellington, New Zealand

**Keywords:** autism, co-occurring conditions, health, health service use, mental health, pharmaceutical dispensing, youth

## Abstract

Many autistic youth have complex healthcare needs, resulting in high rates of health service utilization. However, many characteristics of this health service utilization remain unknown. Using whole-of-population data, this study aimed to quantify and compare rates of psychiatric and non-psychiatric health service utilization among autistic and non-autistic youth, with and without co-occurring intellectual disability. A national retrospective cohort study was employed using linked individual-level administrative data for Aotearoa New Zealand youth (aged 0–24 years). Health service utilization outcomes included 11 psychiatric and non-psychiatric domains. Propensity score matching on a range of sociodemographic measures compared groups across health service utilization outcomes. Data were obtained for 19,479 autistic youth, 29% of whom had a co-occurring intellectual disability and 1,561,278 non-autistic youth. Results demonstrated higher rates of mental health service utilization among autistic compared with non-autistic youth, including inpatient (prevalence ratio 5.85; 95% confidence interval 4.93–6.94) and outpatient (prevalence ratio 4.96; 95% confidence interval 4.75–5.18) service use and psychotropic medication dispensing (prevalence ratio 6.83; 95% confidence interval 6.65–7.02), particularly among autistic youth without intellectual disability. Rates of non-psychiatric hospital admissions (prevalence ratio 1.93; 95% confidence interval 1.85–2.01), potentially avoidable hospitalizations (prevalence ratio 1.91; 95% confidence interval 1.82–2.00) and outpatient visits (prevalence ratio 1.99; 95% confidence interval 1.95–2.01) were also higher among autistic youth. Research implications for delivery of healthcare services are discussed.

**Lay abstract:**

Autistic youth generally use healthcare services more often than non-autistic youth. However, we know very little about the factors that can affect health service use and the types of services that are used, and this has not been explored in Aotearoa New Zealand. We analysed data from New Zealand to compare health service use among autistic and non-autistic youth (0 to 24-year-olds). Data were available for 19,479 autistic youth and 1,561,278 non-autistic youth. We compared hospitalizations, specialist visits, emergency department visits and use of different types of medications. In this study, autistic youth were found to have been hospitalized for medical and mental health reasons, more often than non-autistic youth. Autistic youth were also more likely to have attended specialist appointments and to have been given medication. These differences were particularly large for medications commonly used for mental health conditions (e.g. anxiety, depression, attention deficit hyperactivity disorder) or associated symptoms. Autistic youth who also had an intellectual disability were more likely to use healthcare services for physical health conditions, but were less likely to use mental health services, when compared with autistic youth who did not have an intellectual disability. These findings, along with other research, suggest that the healthcare needs of autistic youth are not always being met. Further work is needed to enhance our understanding of co-occurring conditions among autistic youth, including those that result in high rates of health service use, in order to inform the development of healthcare services and training for healthcare professionals to better cater to the needs of autistic youth.

## Introduction

Autism is a neurodevelopmental condition characterized by differences in social communication and interaction and behaviour (American Psychiatric Association, 2013). In Aotearoa New Zealand (NZ), autism is sometimes referred to as *takiwātanga; a te reo* Māori term meaning ‘in my, his or her own time and space’ (Opai, 2020). In recent years, there has been a global increase in the diagnosis of autism (Maenner et al., 2023; Zeidan et al., 2022). Indeed, data from the United States suggest a greater than four-fold increase in autism diagnoses since 2000 (Maenner et al., 2023). In NZ, current estimates suggest that 1 in 40 children and adolescents (henceforth, youth) are autistic (Ministry of Health, 2023).

Autistic youth have substantially greater mental and physical healthcare needs than their non-autistic counterparts (Bowden, Thabrew, Kokaua, Audas, et al., 2020; Cummings et al., 2016; Lai et al., 2019; Reaven & Wainer, 2015; Rosen et al., 2018). Estimates from a range of cross-sectional, cohort-based and longitudinal studies comprising community-based samples suggest that up to 70% of autistic youth experience co-occurring mental health conditions including anxiety, depression, attention deficit hyperactivity disorder (ADHD) and sleep problems (Bowden, Thabrew, Kokaua, Audas, et al., 2020; Goldman et al., 2012; Hollocks et al., 2023; Malow et al., 2016; Martini et al., 2022; Reaven & Wainer, 2015; Rosen et al., 2018). These rates are far in excess of those typically observed among non-autistic youth. Indeed, estimates suggest that autistic youth are significantly more likely to be diagnosed with anxiety (14.4%–20.6% vs 1.8%–3.8% (autistic vs non-autistic)), depression (4.8%–22.7% vs 1.6%–4.0%), ADHD (14.6%–45.0% vs 0.0%–3.6%), bipolar disorder (1.3%–6.4% vs 0.2%–0.6%), psychosis (1.7%–5.1% vs 0.3%–0.4%) and obsessive-compulsive disorder (2.3%–4.2% vs 0.2%–0.5%) than their non-autistic counterparts (Bowden, Thabrew, Kokaua, Audas, et al., 2020; Davignon et al., 2018; Martini et al., 2022).

A significant proportion of autistic youth also experience co-occurring physical health conditions (Hughes et al., 2018; Lasheras et al., 2023; Liu et al., 2022; McElhanon et al., 2014; Xu et al., 2018). For example, in a medical register-based study comprising demographically matched autistic and non-autistic youth, Davignon et al. (2018) found that autistic youth were significantly more likely to be diagnosed with neurological (17.8% vs 9.2% (autistic vs non-autistic)), autoimmune (16.2% vs 12.5%), gastrointestinal (10.8% vs 8.4%), cardiovascular (6.0% vs 2.6%) and metabolic (3.5% vs 1.0%) disorders. This is similarly identified in meta-analyses, which highlight higher rates of physical health conditions among autistic youth compared with their non-autistic peers (Dhanasekara et al., 2023; Lasheras et al., 2023; McElhanon et al., 2014; Pan et al., 2021). Longitudinal studies underscore the persistent nature of these conditions across childhood and adolescence (Hollocks et al., 2023; Stringer et al., 2020) resulting in lifelong effects on the well-being of autistic youth. This can be perpetuated by inadequate support and environments that are unsafe or not inclusive of the needs of autistic people.

International research consistently reveals elevated levels of healthcare utilization among autistic youth (Barrett et al., 2015; Croen et al., 2006; Lavelle et al., 2014; Schlenz et al., 2015; Weiss et al., 2018). This pattern is observed across a range of services, including engagement with general and specialist outpatient appointments (i.e. paediatric, neurologic and psychiatric; Barrett et al., 2015; Cummings et al., 2016; Lavelle et al., 2014; Rogge & Janssen, 2019; Weiss et al., 2018), therapeutic appointments (e.g. occupational therapy, speech language therapy, psychotherapy; Cummings et al., 2016; Gurney et al., 2006; Peacock et al., 2012), inpatient hospital admissions (Croen et al., 2006; Cummings et al., 2016; Deavenport-Saman et al., 2016; Mandell et al., 2006; Rogge & Janssen, 2019; Schlenz et al., 2015; Weiss et al., 2018) and prescription medication use (Barrett et al., 2015; Bowden, Thabrew, Kokaua, & Braund, 2020; Croen et al., 2006; Lavelle et al., 2014; Liptak et al., 2006; Peacock et al., 2012). In fact, research suggests that autistic youth are between 6 and 12 times as likely to engage with mental health services (e.g. psychiatrist visits, psychiatric inpatient admissions) and psychotropic pharmaceutical dispensing than their non-autistic counterparts (Croen et al., 2006; Schlenz et al., 2015; Weiss et al., 2018). Likewise, findings suggest that autistic youth are significantly more likely to engage with primary and secondary care services for physical health needs (Croen et al., 2006; Schlenz et al., 2015; Weiss et al., 2018). For example, Weiss et al. (2018) revealed that autistic youth were four times more likely to have visited their paediatrician, and nearly seven times more likely to have visited their neurologist relative to non-autistic youth. Evidence suggests that as children age, their engagement with these services tends to increase, primarily driven by the need for management of mental health conditions (Cidav et al., 2013; Peacock et al., 2012; Peng et al., 2009; Rogge & Janssen, 2019).

As a result of increased utilization, data from large nationwide surveys and medical registries suggest that healthcare costs internationally are estimated to be between 2 and 10 times higher among autistic compared with non-autistic youth (Cidav et al., 2013; Croen et al., 2006; Lavelle et al., 2014; Liptak et al., 2006; Peacock et al., 2012; Peng et al., 2009; Rogge & Janssen, 2019). This is widely believed to be the result of increased costs for hospitalizations, medications and outpatient clinic visits, in many cases associated with the management of co-occurring conditions (Croen et al., 2006; Mandell et al., 2006; Peacock et al., 2012; Rogge & Janssen, 2019).

Interestingly, data on emergency department admissions present a more nuanced picture, reflecting variability in rates between autistic and non-autistic youth across studies (Croen et al., 2006; Cummings et al., 2016; Deavenport-Saman et al., 2016; Liptak et al., 2006; Mandell et al., 2006; Weiss et al., 2018). Such variation has been attributed to the nature of the presenting concerns (e.g. mental and/or physical health), the presence of co-occurring physical and mental health conditions (Jain et al., 2014), the type of injury and age categorizations. For example, based on medical registry data for 252 autistic youth and 1260 demographically matched non-autistic youth, Schlenz et al. (2015) found that autistic youth (aged 13–18 years) were more likely to have emergency department admissions for self-inflicted injury, but less likely to have admissions for general injuries/accidents (e.g. motor vehicle or cycling accidents). Likewise, Weiss et al. (2018) found markedly higher odds of psychiatric-related emergency department visits relative to all-cause emergency department visits. Higher rates of emergency department utilization have also been associated with co-occurring ID among autistic young adults (Benevides et al., 2020), demonstrating the need for a broad understanding of the context in which a service is utilized. Collectively, this research suggests that a diagnosis of autism alone does not account for increased emergency department visits, with child characteristics, co-occurrences and injury type playing a potential role.

Effective management and treatment of health conditions is dependent upon engagement with service providers, and the provision of appropriate types and levels of support. In NZ, while criteria are stringently applied, autistic youth can be referred by a general practitioner/primary care physician to publicly available specialist mental health services, with options for parent, self or service provider referral under urgent circumstances. In circumstances in which there is imminent risk of harm to self or others, that is unable to be managed by outpatient services, autistic youth may also be considered for admission to inpatient services. Similarly, to effectively manage and treat mild physical health conditions and/or injuries, individuals in NZ would typically be advised to engage with primary healthcare providers (e.g. general practitioner/primary care physician, registered nurse). However, for specialist care, in the case of severe injury, or when outside of clinic operating hours, people engage with hospital-based or emergency care providers in both inpatient and outpatient settings. In NZ, such healthcare is provided cost free.

Given the high rates of healthcare utilization among autistic youth, it is essential that we enhance our understanding of their healthcare needs and service use patterns. Increased awareness would enable the tailoring of services and supports to best meet the needs of autistic youth; can enable targeted, individualized and appropriate levels of resource allocation; and can support the identification and subsequent reduction of healthcare inequities. Such improvements may enhance the access, effectiveness and experiences of services, including therapeutic approaches (Broder-Fingert et al., 2016; Nicolaidis et al., 2016; Storch et al., 2013, 2015; Wood et al., 2020). In NZ, there are no existing studies that have examined health service utilization (HSU) patterns among autistic youth. Moreover, aside from co-occurring conditions and age-related changes, we know little about the characteristics of high-frequency healthcare users both in NZ and internationally; although there is some evidence of higher healthcare costs (Cidav et al., 2013; Peacock et al., 2012), psychotropic medication prescription (Downs et al., 2016; Zablotsky et al., 2015), and greater unmet mental healthcare needs among autistic children with an intellectual disability (ID; Menezes et al., 2021]). However, further research is needed to corroborate these findings, including examining HSU.

The Integrated Data Infrastructure (IDI) is a research database maintained by Stats NZ that encompasses a wide range of population-level data linked at the individual level (Milne et al., 2019; see ‘Methods’ section). Such data offer a unique opportunity to examine the HSU of autistic youth at a population level, and make comparisons to their non-autistic peers. Therefore, this study aims to use whole-of-population IDI data comparing autistic and non-autistic youth (aged 0–24 years) to quantify (a) inpatient and outpatient mental HSU; (b) hospital admissions for self-harm; (c) pharmaceutical dispensing; (d) inpatient hospitalizations and outpatient presentations, including potentially avoidable hospitalizations and emergency department visits and (e) and variation in HSU for those with and without co-occurring ID.

## Method

### Ethical approval

This study was approved by the University of Otago Human Research Ethics Committee and was reviewed as a ‘Minimal Risk Health Research – Audit and Audit related studies’ proposal and approved (Reference: HD17/004). Stats NZ approved access to the IDI (Reference: MAA2017-16).

### Study design and participants

This was a national retrospective cohort study utilizing individual-level linked administrative data contained within NZ’s IDI. The IDI contains mostly administrative data collected from NZ government agencies such as Manatū Hauora Ministry of Health, Te Tāhuhu o te Mātauranga the Ministry of Education and Ministry of Social Development Te Manatū Whakahiato Ora. These data are collected at a population level when individuals access publicly funded services and are made available within the IDI for research that is deemed to be for the public good. Stats NZ employs probabilistic linkage, typically utilizing name, date of birth and sex to link IDI data at the individual level before subsequently de-identifying it.

The participant population for this study was the IDI estimated resident population for NZ youth (aged 0–24 years) for the 2019 calendar year. This population was constructed using established methods for estimating the estimated resident population within the IDI (Gibb et al., 2016). The chosen age range was selected as it reflects the World Health Organization definition of youth; aligns with the broader programme of research undertaken by the authors; and is the most reliable age range for the autism case identification method employed in this study.

### Health service use

All health outcomes were constructed as binary indicators of publicly funded HSU for the 2019 calendar year.

Publicly funded hospital admissions data contained within the national minimum dataset (NMDS) were used to establish indicators for non-psychiatric hospital inpatient stays, potentially avoidable hospitalizations (PAH; i.e. hospitalizations that could have been prevented or avoided) and admissions for self-harm. Hospital inpatient stays included all inpatient events contained within NMDS but excluded mental health-related events based on health specialty coding, a classification describing the specialty or service that provided the health care for the individual. The PAH indicator was constructed using an established Ministry of Health methodology for PAH among children and young people (Ministry of Health, 2020). PAH include admissions for respiratory conditions, dental conditions, gastrointestinal diseases, nutrition deficiency and anaemia, cardiovascular diseases, otitis media, dermatological conditions, diabetes complications, kidney, urinary tract infection, sexually transmitted infections, vaccine-preventable diseases, meningococcal infection, epilepsy, other non-injury conditions, unintentional injuries and intentional injuries. Self-harm events were indicated if an individual was admitted to hospital with an ICD-10-AM code for intentional self-harm (X60-X84 or Y870), which include self-inflicted poisoning and injuries.

The national non-admitted patient collection (NNPAC) was used to indicate ED presentations and outpatient service use.

The Programme for the Integration of Mental Health Data (PRIMHD), a dataset containing publicly funded specialist mental health service use information, was used to indicate psychiatric inpatient admissions and specialty mental health outpatient service use.

The pharmaceutical collection, a national collection of all government subsidized medication dispensing from community pharmacies, was used to indicate psychotropic and non-psychotropic pharmaceutical dispensing. Categorization of medications into psychotropic and non-psychotropic drew on the groupings constructed by McLay et al. (2022).

Enrolment with a general practice and primary health organization (PHO) was determined using the PHO Enrolment Collection. An individual was considered to be enrolled with a PHO if they were listed in the PHO Enrolment Collection at any time in the 2019 calendar year.

Accident Compensation Corporation (ACC) support data were used to identify individuals who had received support through either income compensation or having their medical expenses paid for by ACC. ACC is a comprehensive no-fault system that covers all individuals who sustain injuries in accidents that occur within NZ.

### Autism

The identification of the autistic population employed a well-established case identification method, as outlined in Bowden, Thabrew, Kokaua, Audas, et al. (2020). This method leverages diagnostic information extracted from three national health datasets: NMDS, PRIMHD and Socrates, a national collections of disability support services. Autism status was determined if an autism diagnosis code was present in any of the three datasets from birth (refer to Table 1 in Supplementary Material for further details). In cases where no autism diagnosis code was identified, individuals within the cohort were categorized into the non-autistic (general population) group. It is important to note that due to the reliance on HSU and recorded diagnoses, coupled with the absence of data from primary care and outpatient settings, this method is anticipated to underestimate the prevalence of autism cases (Bowden, Thabrew, Kokaua, Audas, et al., 2020).

### ID

The process for identifying co-occurring ID mirrored that of autism identification and was likewise grounded in a well-established method (Bowden, Thabrew, Kokaua, Audas, et al., 2020). Within the autistic population, diagnostic information extracted from PRIMHD, NMDS and Socrates was examined, and the presence of ID was determined if an ID code was identified in any of these datasets (refer to Table 2 in Supplementary Material for additional information).

### Sociodemographic variables

Sex (male/female), age (0–4, 5–9, 10–14, 15–19 and 20–24 years) and ethnicity were obtained from the IDI personal details table. Sex was categorized as male/female due to data being unavailable for non-binary identities. Age was determined as at 31 December 2019. Ethnicity was determined using the total concept approach, whereby individuals can identify with multiple ethnic groups. We categorized individuals as Māori, Pacific and non-Māori/non-Pacific (NMNP) using three separate binary indicators. Area-level deprivation and urban/rural classification of residence were determined by extracting residence data from the address notification table, measured at 31 December 2019. Deprivation was classified using the NZ Index of Deprivation, 2018 (NZDep) (Atkinson et al., 2020). NZDep is an area-level deprivation measure (based on areas of approximately 100–200 residents), consolidating census information on various domains, such as employment, income, education level and home ownership into a single continuous index. For this study, the index was condensed into quintiles, with one denoting the least deprived and five signifying the highest level of deprivation. Utilizing Stats NZ’s urban/rural classification, we established a binary urban/rural measure. Rural areas represent those with populations of fewer than 1000 people.

### Statistical analysis

Observed rates of health outcomes were presented for autistic and non-autistic populations, and for the autistic population, stratified by ID status. To compare outcomes across groups, matched samples of non-autistic youth were drawn from the IDI estimated resident population of 0 to 24-year-olds for 2019 for the autistic, autistic without ID and autistic with ID populations. A propensity score matching approach was employed to select the matched non-autistic comparison groups. Matching was undertaken using the ‘MatchIt’ package version 3.0.2 in R using nearest neighbour 1-to-10 matching. Matches were drawn without replacement from the IDI estimated resident population for 2019 on age, sex, ethnicity, deprivation and urban/rural profile of residence. Individuals who had never had an autism diagnosis or had missing data on any of the matching variables were not eligible to be selected as matches. For the matched samples, rates of HSU and associated prevalence ratios (PRs) and 95% confidence intervals (CIs) were presented. Reporting of results was informed by the RECORD guidelines (Benchimol et al., 2015).

### Community involvement

Our research actively involves the autistic community (i.e. autistic individuals) and the broader autism community (e.g. parents, caregivers, family members and professionals regularly supporting autistic individuals). One of the co-authors brings the perspective of being an autistic adult, a mother to an autistic adult son and a grandmother to an autistic child. She actively engages in advocacy roles, representing both the autistic and autism communities. The authorship team also includes an autistic researcher and two clinicians who work closely with autistic children, their families and communities.

## Results

### Participant characteristics

The 2019 NZ IDI estimated resident population contained 1,580,757 eligible youth, 19,479 (1.2%) of whom were identified as autistic. Among the autistic youth population 5658 (29.0%) were identified with a co-occurring ID. The sociodemographic characteristics of the participant population, stratified by autism and ID status, are presented in Table 1. The autistic population was more likely to be male and less likely to identify as either Māori or Pacific, or to live in rural areas compared with the non-autistic population. Among the autistic population, those with a co-occurring ID were more likely to identify as Māori or Pacific, live in the most deprived areas, and less likely to live in rural areas, compared with autistic youth without an ID.

**Table 1.** Population demographics of eligible cohort by autism status (and by ID status within the autism group), 2019.

	Autistic	Non-autistic	Autistic without ID	Autistic with ID
	*n* (%)	*n* (%)	*n* (%)	*n* (%)
Total	19,479	1,561,278	13,821	5658
Sex				
Female	4227 (21.7)	763,959 (48.9)	2925 (21.2)	1302 (23.0)
Male	15,252 (78.3)	797,319 (51.1)	10,896 (78.8)	4356 (77.0)
Age (years)				
0–4	2064 (10.6)	293,046 (18.8)	1380 (10.0)	684 (12.1)
5–9	4998 (25.7)	314,592 (20.1)	3513 (25.4)	1485 (26.2)
10–14	5172 (26.6)	317,661 (20.3)	3777 (27.3)	1395 (24.7)
15–19	4221 (21.7)	306,360 (19.6)	3021 (21.9)	1200 (21.2)
20–24	3027 (15.5)	329,622 (21.1)	2130 (15.4)	897 (15.9)
Ethnicity^a^				
Māori	4701 (24.1)	406,230 (26.0)	3210 (23.2)	1491 (26.4)
Pacific	1944 (10.0)	214,530 (13.7)	1029 (7.4)	915 (16.2)
Non-Māori/non-Pacific	13,563 (69.6)	999,009 (64.0)	10,008 (72.4)	3555 (62.8)
Deprivation quintile^b^				
1 (least deprived)	3678 (19.2)	313,017 (20.4)	2724 (20.1)	954 (17.1)
2	3639 (19.0)	289,554 (18.9)	2634 (19.4)	1005 (18.0)
3	3714 (19.4)	285,627 (18.7)	2754 (20.3)	960 (17.2)
4	3894 (20.3)	293,619 (19.2)	2796 (20.6)	1098 (19.7)
5 (most deprived)	4224 (22.1)	349,518 (22.8)	2670 (19.7)	1554 (27.9)
Urban/rural^c^				
Urban	17,103 (89.1)	1,339,074 (87.2)	12,033 (88.4)	5070 (91.0)
Rural	2082 (10.9)	196,182 (12.8)	1578 (11.6)	504 (9.0)

a In New Zealand, it is possible to identify with multiple ethnic groups and therefore percentages will sum to over 100%.

b Missing data for 11,124 (0.7%) individuals.

c Missing data for 7131 (0.5%) individuals.

### Propensity score matched health outcomes between groups

The matched comparison groups arising from the 1:10 propensity score matching approach had equal distributions across age, sex, ethnicity, deprivation and urbanicity (see Table 3 in the Supplementary Material).

Table 2 displays propensity score HSU rates and associated PRs and 95% CIs for the autistic population compared with the non-autistic population (for observed crude HSU rates for the full participant population refer to Table 4 in the Supplementary Material). Overall, the autistic population experienced higher rates of HSU compared with their matched non-autistic counterparts across all outcomes except ACC claims. HSU was particularly high among the autistic population for mental health-related care including inpatient admissions (PR, 5.85; 95% CI, 4.93–6.94), outpatient visits (PR, 4.96; 95% CI, 4.75–5.18) and psychotropic medication dispensing (PR, 6.83; 95% CI, 6.65–7.02). Hospital presentations for self-harm were also significantly higher among the autistic population (PR, 3.91; 95% CI, 3.19–4.79). In addition, the autistic population was also significantly more likely to be admitted to hospital for non-psychiatric treatment (PR, 1.93; 95% CI, 1.85–2.01), experience a PAH (PR, 1.91; 95% CI, 1.82–2.00) and have an outpatient visit (PR, 1.99; 95% CI, 1.95–2.01). In contrast, rates of ACC claims among the autistic population were 25% lower (PR, 0.75; 95% CI, 0.73–0.77) compared with non-autistic youth.

**Table 2.** Health service use comparison between 1:10 propensity score matched autistic (*N* = 19,149) and non-autistic populations (*N* = 191,490).

	Autistic	Non-autistic	Autistic/non-autistic
	*n* (%)	*n* (%)	PR (95% CI)
Hospitalization			
Yes	2397 (12.5)	12,408 (6.5)	1.93 (1.85, 2.01)
No	16,755 (87.5)	179,082 (93.5)	
Potentially avoidable hospitalization			
Yes	1797 (9.4)	9423 (4.9)	1.91 (1.82, 2.00)
No	173,55 (90.6)	182,067 (95.1)	
Self-harm			
Yes	129 (0.7)	330 (0.2)	3.91 (3.19, 4.79)
No	19,023 (99.3)	191,160 (99.8)	
Emergency department			
Yes	3315 (17.3)	29,454 (15.4)	1.13 (1.09, 1.16)
No	15,837 (82.7)	162,036 (84.6)	
Outpatient			
Yes	9963 (52.0)	50,331 (26.3)	1.98 (1.95, 2.01)
No	9189 (48.0)	141,159 (73.7)	
Psychiatric inpatient			
Yes	207 (1.1)	354 (0.2)	5.85 (4.93, 6.94)
No	18,945 (98.9)	191,136 (99.8)	
Psychiatric outpatient			
Yes	2850 (14.9)	5745 (3.0)	4.96 (4.75, 5.18)
No	16,302 (85.1)	185,745 (97.0)	
Psychotropic pharmaceutical dispensing			
Yes	6813 (35.6)	9969 (5.2)	6.83 (6.65, 7.02)
No	12,339 (64.4)	181,521 (94.8)	
Non-psychotropic pharmaceutical dispensing			
Yes	14,028 (73.3)	127,944 (66.8)	1.1 (1.09, 1.11)
No	5124 (26.8)	63,546 (33.2)	
Primary health care enrolment			
Yes	18,960 (99.0)	186,390 (97.3)	1.02 (1.02, 1.02)
No	192 (1.0)	5100 (2.7)	
Accident compensation scheme			
Yes	4527 (23.6)	60,717 (31.7)	0.75 (0.73, 0.77)
No	14,625 (76.4)	130,773 (68.3)	

PAH: respiratory conditions, dental conditions, gastrointestinal diseases, nutrition deficiency and anaemia, cardiovascular diseases, otitis media, dermatological conditions, diabetes complications, kidney, urinary tract infection, sexually transmitted infections, vaccine-preventable diseases, meningococcal infection, epilepsy, other non-injury conditions, unintentional injuries, intentional injuries.

The HSU patterns for the autistic population with and without co-occurring ID, compared with their matched non-autistic populations were similar to those of the collective autistic population (see Table 3). Rates of HSU were higher among autistic youth with and without ID, compared with the matched non-autistic population, for all domains except ACC support. Relative rates of HSU were particularly high for mental health care including psychiatric inpatient hospitalisations (PR, 6.02; 95% CI, 4.92–7.37 (autistic without ID) and PR, 6.13; 95% CI, 4.41–8.51 (autistic with ID)), psychiatric outpatient visits (PR, 5.35; 95% CI, 5.09–5.62 (autistic without ID) and PR, 3.96; 95% CI, 3.63–4.31 (autistic with ID)), and psychotropic medication dispensing (PR, 6.60; 95% CI, 6.39–6.82 (autistic without ID) and PR, 7.34; 95% CI, 6.98–7.71 (autistic with ID)). Rates of ACC use were 24% and 28% lower among autistic youth without and with ID, compared with their non-autistic counterparts (PR, 0.76; 95% CI, 0.73–0.78 (autistic without ID) and PR, 0.72; 95% CI, 0.68–0.75 (autistic with ID)).

**Table 3.** Health service use comparison between the autistic population without ID (*N* = 13,578) and the 1:10 propensity score matched non-autistic population (*N* = 135,780), and the autistic population with ID (*N* = 5571) and the 1:10 propensity score matched non-autistic populations (*N* = 55,710).

	Autistic without ID	Non-autistic	Autistic without ID/non-autistic	Autistic with ID	non-autistic	Autistic with ID/non-autistic
	*n* (%)	*n* (%)	PR (95% CI)	*n* (%)	*n* (%)	PR (95% CI)
Hospitalization						
Yes	1461 (10.8)	8724 (6.4)	1.67 (1.59, 1.76)	933 (16.7)	3729 (6.7)	2.50 (2.34, 2.67)
No	12,117 (89.2)	127,056 (93.6)		4638 (83.3)	51,981 (93.3)	
Potentially avoidable hospitalization						
Yes	1122 (8.3)	6519 (4.8)	1.72 (1.62, 1.83)	675 (12.1)	2913 (5.2)	2.32 (2.14, 2.51)
No	124,56 (91.7)	129,261 (95.2)		4896 (87.9)	52,797 (94.8)	
Self-harm						
Yes	108 (0.8)	243 (0.2)	4.44 (3.55, 5.57)	21 (0.4)	84 (0.2)	2.50 (1.55, 4.03)
No	134,70 (99.2)	135,537 (99.8)		5,550 (99.6)	55,626 (99.8)	
Emergency department						
Yes	2310 (17.0)	20,502 (15.1)	1.13 (1.08, 1.17)	1008 (18.1)	8958 (16.1)	1.13 (1.06, 1.19)
No	11,268 (83.0)	115,278 (84.9)		4563 (81.9)	46,752 (83.9)	
Outpatient						
Yes	6702 (49.4)	35,916 (26.5)	1.87 (1.83, 1.90)	3261 (58.5)	14,391 (25.8)	2.27 (2.21, 2.33)
No	6876 (50.6)	99,864 (73.5)		2310 (41.5)	41,319 (74.2)	
Psychiatric inpatient						
Yes	150 (1.1)	249 (0.2)	6.02 (4.92, 7.37)	57 (1.0)	93 (0.2)	6.13 (4.41, 8.51)
No	13,428 (98.9)	135,531 (99.8)		5514 (99.0)	55,617 (99.8)	
Psychiatric outpatient						
Yes	2187 (16.1)	4089 (3.0)	5.35 (5.09, 5.62)	660 (11.8)	1668 (3.0)	3.96 (3.63, 4.31)
No	11,391 (83.9)	131,691 (97.0)		4911 (88.2)	54,042 (97.0)	
Psychotropic pharmaceutical dispensing						
Yes	4752 (35.0)	7200 (5.3)	6.60 (6.39, 6.82)	2061 (37.0)	2808 (5.0)	7.34 (6.98, 7.71)
No	8826 (65.0)	128,580 (94.7)		3510 (63.0)	52,902 (95.0)	
Non-psychotropic pharmaceutical dispensing						
Yes	9729 (71.7)	90,255 (66.5)	1.08 (1.07, 1.09)	4299 (77.2)	37,719 (67.7)	1.14 (1.12, 1.16)
No	3849 (28.3)	45,525 (33.5)		1272 (22.8)	17,991 (32.3)	
Primary healthcare enrolment						
Yes	13,443 (99.0)	132,093 (97.3)	1.02 (1.02, 1.02)	5517 (99.0)	54,363 (97.6)	1.01 (1.01, 1.02)
No	135 (1.0)	3687 (2.7)		54 (1.0)	1347 (2.4)	
Accident compensation scheme						
Yes	3288 (24.2)	43,431 (32)	0.76 (0.73, 0.78)	1242 (22.3)	17,358 (31.2)	0.72 (0.68, 0.75)
No	10,290 (75.8)	92,349 (68)		4329 (77.7)	38,352 (68.8)	

PAH: respiratory conditions, dental conditions, gastrointestinal diseases, nutrition deficiency and anaemia, cardiovascular diseases, otitis media, dermatological conditions, diabetes complications, kidney, urinary tract infection, sexually transmitted infections, vaccine-preventable diseases, meningococcal infection, epilepsy, other non-injury conditions, unintentional injuries, intentional injuries.

### Comparison of PRs across all groups

**Figure 1.**
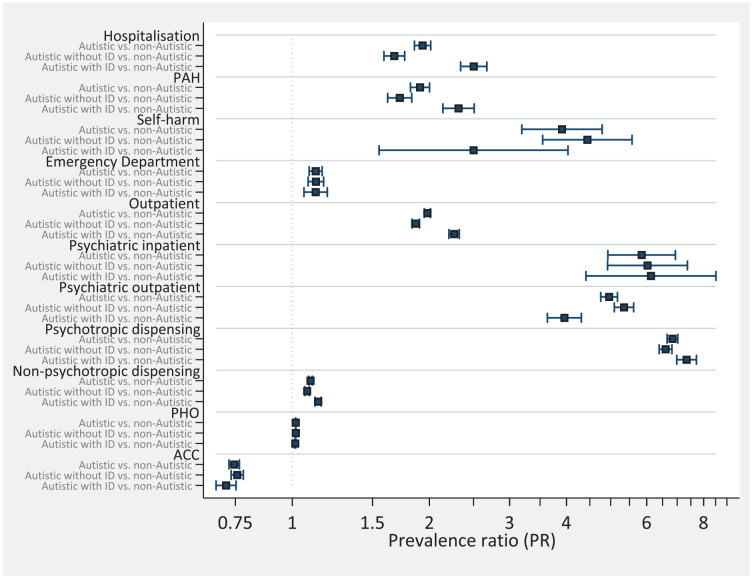
Propensity score matched rate ratios comparing autistic, autistic without ID and autistic with ID to matched non-autistic samples. PAH: respiratory conditions, dental conditions, gastrointestinal diseases, nutrition deficiency and anaemia, cardiovascular diseases, otitis media, dermatological conditions, diabetes complications, kidney, urinary tract infection, sexually transmitted infections, vaccine-preventable diseases, meningococcal infection, epilepsy, other non-injury conditions, unintentional injuries, intentional injuries; ID: intellectual disability; PHO: Primary Health Organizations; ACC: Accident Compensation Corporation.

Figure 1 presents PRs for each domain of HSU between each of the three autistic populations compared with their matched non-autistic counterparts. It demonstrates that autistic youth with a co-occurring ID had substantially higher PRs for several non-psychiatric domains of HSU including inpatient hospitalizations, PAHs, and outpatient visits compared with autistic youth without an ID. In contrast, PRs for several domains of psychiatric and related care were lower for autistic youth with a co-occurring ID including psychiatric outpatient visits and hospital admissions for self-harm compared with autistic youth without an ID.

The HSU patterns by demographic sub-groups (i.e. sex, age, ethnicity, deprivation level and urbanicity) for the autistic and non-autistic population are described in Supplementary Tables 6–16.

## Discussion

This nationwide whole-of-population study is the first to compare inpatient and outpatient mental health service use, hospital admissions, outpatient presentations, and pharmaceutical dispensing among autistic and non-autistic youth in NZ. Furthermore, it is one of few studies internationally, to have examined variation in healthcare utilization according to co-occurring ID. With the exception of ACC claims (i.e. claims for accidental injuries), findings reflect higher observed rates of healthcare utilization among autistic youth.

As evident from international research (Barrett et al., 2015; Croen et al., 2006; Cummings et al., 2016; Gurney et al., 2006; Lavelle et al., 2014; Liptak et al., 2006; Peacock et al., 2012), Autistic youth in NZ had substantially higher rates of mental health service use, psychotropic medication dispensing and self-harm presentation compared with their non-autistic peers, suggesting higher mental health support needs among autistic youth. In the present study, these rates were observed to be lower among autistic youth with an ID, compared with autistic youth without ID, particularly with regard to outpatient psychiatric service use and self-harm presentations. Conversely, autistic youth with an ID were more likely to have received psychotropic medication than those without an ID.

Collectively, these findings highlight high levels of mental health service use among autistic youth. Evidence-based, non-medical approaches to the management of mental health conditions typically include behavioural and cognitive therapies (Åslund et al., 2018; Riise et al., 2021; Sigurvinsdóttir et al., 2020; Zhou et al., 2020). However, these have primarily been designed for neurotypical youth and may have limited potential benefit for autistic people when delivered in the traditional form or by less experienced therapists, or not even be offered to this group on the assumption that their cognitive and communication needs will render them ineffective (Cooper et al., 2018). Given the high rates of mental health service use and psychotropic pharmacotherapy among autistic youth, further research is needed that evaluates the design and delivery of evidence-based non-medical therapies for autistic youth, with and without ID, in order to provide them with autonomy and choice about the types of treatments and supports that they receive and to ensure access to evidence-based support.

Access to outpatient psychiatric services, including for self-harm, may also be contingent upon a person’s ability to communicate their mental health difficulties, as well as professionals’ ability to identify and respond to the mental health challenges in those with complex communication or cognitive needs. Moreover, owing to their developmental stage, many autistic youth are dependent upon their caregivers to access necessary support, many of whom may be unaware of the high rates of co-occurring psychiatric conditions, and the symptom of these conditions, in autistic youth. This combination of factors ultimately affects both service access and scope. Further attention is needed to accurately identify and assess mental health difficulties among those with complex communication needs.

In addition to psychiatric services, data suggest that autistic youth were more likely to be admitted to hospital for non-psychiatric care; were more likely to experience potentially avoidable hospitalizations; and, albeit to a lesser extent, had higher rates of ED visits. Likewise, engagement with outpatient support, including use of non-psychotropic medications and enrolment with a PHO was slightly elevated among autistic compared with non-autistic youth. Unlike psychiatric service use, autistic youth with an ID had substantially higher rates of non-psychiatric service use compared with autistic youth without an ID, across inpatient and outpatient services, particularly, those resulting in hospitalization. Moreover, they were more likely to be dispensed non-psychiatric medications compared with autistic youth without an ID.

The higher relative rates of non-psychiatric healthcare utilization are well documented internationally (Croen et al., 2006; Cummings et al., 2016; Deavenport-Saman et al., 2016; Mandell et al., 2006; Rogge & Janssen, 2019; Schlenz et al., 2015; Weiss et al., 2018), and may reflect higher rates and a greater severity of co-occurring physical health conditions compared with non-autistic youth (Cummings et al., 2016; Davignon et al., 2018; Kohane et al., 2012). This may in part be attributed towards an increased likelihood of co-occurring syndromic diagnoses among autistic youth (Cummings et al., 2016; Davignon et al., 2018; Genovese & Butler, 2024), many of which are accompanied by co-occurring physical health conditions and/or ID. Likewise, it may suggest greater difficulty communicating physical symptoms (Babalola et al., 2024), missing critical opportunities for early treatment and increasing reliance on acute delivery of healthcare services. Higher rates of non-psychiatric healthcare utilization among autistic youth with an ID, most strikingly in relation to inpatient hospitalizations and PAH, has scarcely been explored previously, though there is evidence to suggest that those with an ID have more complex physical healthcare needs than those without an ID (Etyemez et al., 2022; Liu et al., 2022; Vidriales-Fernández et al., 2023), and thus, require higher levels and greater consistency of medical care. Moreover, those with an ID may experience increased difficulty recognizing, understanding and communicating symptoms of their health conditions that could enable preventive care or otherwise mitigate an escalation in symptoms. Relatedly, those with an ID may require greater support maintaining management and treatment regimens for respective physical health conditions, resulting in needs for higher levels and/or adapted models of care (Haller et al., 2022; Menezes et al., 2021).

Given the higher rates of healthcare utilization, it is difficult to explain why the rates of engagement with ACC are lower among autistic compared with non-autistic youth. It is possible that this may reflect lower rates of unintentional injury (Jain et al., 2014; Jernbro et al., 2020) or injuries that meet criteria for ACC funding. It is also possible that this may reflect barriers to accessing ACC, including difficulty navigating the systems and processes necessary to enable access and self-advocacy, structural barriers such as difficulty travelling to ACC appointments and associated time commitments, reluctance to share sensitive issues with unfamiliar people, or difficulty understanding eligibility criteria. Many such barriers can be experienced directly by youth, or by their wider support network who often assume responsibility for navigating healthcare systems on behalf of autistic youth. Conversely, it may reflect the biases or a limited understanding of autism among those assessing claims from autistic people. Further research is needed to understand how autistic youth can be supported to access such vital services, while simultaneously considering how staff may better understand and respond to the enablers and barriers to access experienced by autistic youth.

These findings have several implications for design and delivery of healthcare services for autistic youth. Despite consistent evidence demonstrating higher rates of healthcare utilization among autistic compared with non-autistic youth, healthcare services continue to be designed based on neurotypical, Western models of care. While current models more adequately meet the needs of the majority population, to address healthcare inequities among marginalized groups, including multiply divergent individuals, it is critical to consider alternative or adjunct models. Co-design of such services, in this case, with autistic youth and their families, would go a long way to enhancing the suitability of services and supports and addressing barriers to accessing primary healthcare services. Further public education and training focused on the healthcare needs of autistic youth, including the identification, assessment and management of co-occurring conditions is needed, particularly for autistic youth with an ID. This should include increased education and training relating to diagnostic overshadowing, which presents a common barrier to accessing necessary support among autistic youth (Christou, 2016; Gupta & Gupta, 2023). Further education and training for both healthcare providers, including those in primary healthcare, and caregivers and others supporting autistic youth about non-verbal signs of psychological or physical distress and/or mental health difficulties also need to be developed. Finally, for autistic youth themselves it is important to provide a means for them to recognize and communicate health symptoms; to advocate for their healthcare needs; and to manage treatment regimens for existing conditions. However, this must go hand in hand with healthcare practitioners presuming competence and providing developmentally appropriate levels of autonomy for autistic people to be fully involved in decisions relating to their care.

The findings of this study must be considered in light of several limitations. The method employed for identifying the autistic population, while used extensively in existing literature (Bowden et al., 2022; McLay et al., 2022; Vu et al., 2024), has yet to be validated. Therefore, the extent to which it undercounts true cases of autism, or identifies false positives remains unknown. While it is not possible to be definitive, based on existing evidence, the method likely undercounts the underlying prevalence of autism (Bowden, Thabrew, Kokaua, Audas, et al., 2020), and particularly so among marginalized groups (e.g. ethnic minorities) such as Pacific populations (Ruhe et al., 2022). In addition, while the propensity score matching controlled for a range of sociodemographic characteristics, other potentially influential measures such as age at diagnosis, co-occurring conditions and familial variables were not accounted for. Data on non-binary identities were also unavailable. It is also noteworthy that while the medications identified in this study are typically used to manage mental health conditions, it is possible that these medications were used for a wider range of purposes for autistic youth (e.g. management of autism-related symptoms). In addition, engagement with each health service was dichotomized meaning that there was no differentiation between those who had single versus multiple interactions with health services. As such, HSU burden will be underestimated in this study. Finally, while the study examined a range of domains of HSU, there are some notable areas in which data were not available. For example, the IDI does not contain detailed information from primary care. Therefore, beyond enrolment with a general practitioner, we were not able to examine health services provided in this setting. Likewise, privately funded health care, such as private community paediatric care, while relatively uncommon in NZ, is not captured in the IDI and therefore not included in the study.

These findings highlight a number of avenues for further research. First, future research should enhance our understanding of the specific physical and mental health conditions, or other causes of healthcare utilization (e.g. reasons for ACC claims, primary cause of inpatient and outpatient service use) and the specific types of HSU within each of the categories applied in this study. Second, we must better understand the demographic characteristics of high users of healthcare services, including how service use differs across the early life course and the service use patterns and need of autistic Māori who are likely to face additional and compounded barriers to accessing high-quality, culturally responsive healthcare services. Third, we must develop a clearer understanding of trends in healthcare utilization by age and among autistic and non-autistic youth with ID. Fourth, we need to establish a clearer understanding of the enablers and barriers to accessing healthcare services and supports, including primary healthcare and post-illness or injury supports (e.g. ACC). Fifth, it is important for further research to explore nuanced patterns of healthcare use that reflect repeated presentations to health services. Collectively, this will enable more nuanced, efficient and effective planning, design and delivery of healthcare services for autistic youth.

## Conclusion

These findings highlight the complex healthcare needs of autistic youth and the importance of ensuring that our healthcare systems are appropriately designed and equipped to provide comprehensive and accessible care to meet these needs. Addressing barriers to accessing primary and preventive care, and providing autism-appropriate support, may mitigate hospitalizations and outpatient service use, while enhancing the health and well-being of autistic youth.

## Supplemental Material

sj-docx-1-aut-10.1177_13623613241298352 – Supplemental material for Health service utilization among autistic youth in Aotearoa New Zealand: A nationwide cross-sectional study Supplemental material, sj-docx-1-aut-10.1177_13623613241298352 for Health service utilization among autistic youth in Aotearoa New Zealand: A nationwide cross-sectional study by Laurie K McLay, Philip J Schluter, John Williams, Francesca Anns, Ruth Monk, Joanne Dacombe, Gabrielle Hogg, Jessica Tupou, Troy Ruhe, Taylor Scott, Emma Woodford, Hiran Thabrew and Nicholas Bowden in Autism

sj-docx-2-aut-10.1177_13623613241298352 – Supplemental material for Health service utilization among autistic youth in Aotearoa New Zealand: A nationwide cross-sectional study Supplemental material, sj-docx-2-aut-10.1177_13623613241298352 for Health service utilization among autistic youth in Aotearoa New Zealand: A nationwide cross-sectional study by Laurie K McLay, Philip J Schluter, John Williams, Francesca Anns, Ruth Monk, Joanne Dacombe, Gabrielle Hogg, Jessica Tupou, Troy Ruhe, Taylor Scott, Emma Woodford, Hiran Thabrew and Nicholas Bowden in Autism

sj-docx-3-aut-10.1177_13623613241298352 – Supplemental material for Health service utilization among autistic youth in Aotearoa New Zealand: A nationwide cross-sectional study Supplemental material, sj-docx-3-aut-10.1177_13623613241298352 for Health service utilization among autistic youth in Aotearoa New Zealand: A nationwide cross-sectional study by Laurie K McLay, Philip J Schluter, John Williams, Francesca Anns, Ruth Monk, Joanne Dacombe, Gabrielle Hogg, Jessica Tupou, Troy Ruhe, Taylor Scott, Emma Woodford, Hiran Thabrew and Nicholas Bowden in Autism

sj-docx-4-aut-10.1177_13623613241298352 – Supplemental material for Health service utilization among autistic youth in Aotearoa New Zealand: A nationwide cross-sectional study Supplemental material, sj-docx-4-aut-10.1177_13623613241298352 for Health service utilization among autistic youth in Aotearoa New Zealand: A nationwide cross-sectional study by Laurie K McLay, Philip J Schluter, John Williams, Francesca Anns, Ruth Monk, Joanne Dacombe, Gabrielle Hogg, Jessica Tupou, Troy Ruhe, Taylor Scott, Emma Woodford, Hiran Thabrew and Nicholas Bowden in Autism

sj-docx-5-aut-10.1177_13623613241298352 – Supplemental material for Health service utilization among autistic youth in Aotearoa New Zealand: A nationwide cross-sectional study Supplemental material, sj-docx-5-aut-10.1177_13623613241298352 for Health service utilization among autistic youth in Aotearoa New Zealand: A nationwide cross-sectional study by Laurie K McLay, Philip J Schluter, John Williams, Francesca Anns, Ruth Monk, Joanne Dacombe, Gabrielle Hogg, Jessica Tupou, Troy Ruhe, Taylor Scott, Emma Woodford, Hiran Thabrew and Nicholas Bowden in Autism
