# Supplementary material for: Health service utilization among autistic youth in Aotearoa New Zealand: A nationwide cross-sectional study
Source: Autism. 2024 Dec 3;29(5):1143–56. doi: 10.1177/13623613241298352 (PMC12038068; doi:10.1177/13623613241298352)
Supplement: sj-docx-1-aut-10.1177_13623613241298352 – Supplemental material for Health service utilization among autistic youth in Aotearoa New Zealand: A nationwide cross-sectional study [file sj-docx-1-aut-10.1177_13623613241298352.docx]

Supplementary Table 1: Diagnostic codes for identifying autism

| **Dataset** | **Code Type** | **Code** | **Code Description** |
| --- | --- | --- | --- |
| NMDS & PRIMHD | ICD-10-AM | F84.0 | Autistic disorder |
| NMDS & PRIMHD | ICD-10-AM | F84.1 | Atypical autism |
| NMDS & PRIMHD | ICD-10-AM | F84.3 | Other childhood disintegrative disorder |
| NMDS & PRIMHD | ICD-10-AM | F84.5 | Asperger’s syndrome |
| NMDS & PRIMHD | ICD-10-AM | F84.8 | Other pervasive developmental disorders |
| NMDS & PRIMHD | ICD-10-AM | F84.9 | Pervasive developmental disorder, unspecified |
| PRIMHD | DSM-IV | 299.00 | Autistic disorder |
| PRIMHD | DSM-IV | 299.10 | Other childhood disintegrative disorder |
| PRIMHD | DSM-IV | 299.80 | Asperger’s disorder/pervasive development disorder NOS |
| Socrates | Assigned Diagnosis | 1206 | Asperger’s syndrome |
| Socrates | Assigned Diagnosis | 1207 | Other ASD |
| Socrates | Assigned Diagnosis | 1211 | Autism spectrum disorder |

NMDS – National Minimum Dataset

PRIMHD – Programme for the Integration of Mental Health Data

ICD-10-AM – International Statistical Classification of Diseases and Related Health Problems, Tenth Revision, Australian Modification

DSM-IV – Diagnostic and Statistical Manual of Mental Disorders, 4^th^ edition
