# Supplementary material for: Health service utilization among autistic youth in Aotearoa New Zealand: A nationwide cross-sectional study
Source: Autism. 2024 Dec 3;29(5):1143–56. doi: 10.1177/13623613241298352 (PMC12038068; doi:10.1177/13623613241298352)
Supplement: sj-docx-2-aut-10.1177_13623613241298352 – Supplemental material for Health service utilization among autistic youth in Aotearoa New Zealand: A nationwide cross-sectional study [file sj-docx-2-aut-10.1177_13623613241298352.docx]

Supplementary Table 2: Diagnostic codes for identifying ID

| Dataset | Code Type | Code | Code Description |
| --- | --- | --- | --- |
| NMDS & PRIMHD | ICD-10-AM | F70.x | Mild mental retardation |
| NMDS & PRIMHD | ICD-10-AM | F71.x | Moderate mental retardation |
| NMDS & PRIMHD | ICD-10-AM | F72.x | Severe mental retardation |
| NMDS & PRIMHD | ICD-10-AM | F73.x | Profound mental retardation |
| NMDS & PRIMHD | ICD-10-AM | F78.x | Other mental retardation |
| NMDS & PRIMHD | ICD-10-AM | F79.x | Unspecified mental retardation |
| PRIMHD | DSM-IV | 317.x | Mild mental retardation |
| PRIMHD | DSM-IV | 318.0 | Moderate mental retardation |
| PRIMHD | DSM-IV | 318.1 | Severe mental retardation |
| PRIMHD | DSM-IV | 318.2 | Profound mental retardation |
| PRIMHD | DSM-IV | 319.x | mental retardation, severity unspecified |
| PRIMHD | Team Type | 12 | Intellectual Disability Dual Diagnosis Team |
| Socrates | Assigned Diagnosis | 1208 | Intellectual disability, type not specified |
| Socrates | Assigned Diagnosis | 1209 | Learning disability, type not specified |
| Socrates | Assigned Diagnosis | 1210 | Developmental delay, type not specified |
| Socrates | Assigned Diagnosis | 1299 | Other intellectual, learning or developmental disorder |

NMDS – National Minimum Dataset

PRIMHD – Programme for the Integration of Mental Health Data

ICD-10-AM – International Statistical Classification of Diseases and Related Health Problems, Tenth Revision, Australian Modification

DSM-IV – Diagnostic and Statistical Manual of Mental Disorders, 4^th^ edition

Team Type – A code which described the team who provided a service
