# Supplementary material for: Health service utilization among autistic youth in Aotearoa New Zealand: A nationwide cross-sectional study
Source: Autism. 2024 Dec 3;29(5):1143–56. doi: 10.1177/13623613241298352 (PMC12038068; doi:10.1177/13623613241298352)
Supplement: sj-docx-3-aut-10.1177_13623613241298352 – Supplemental material for Health service utilization among autistic youth in Aotearoa New Zealand: A nationwide cross-sectional study [file sj-docx-3-aut-10.1177_13623613241298352.docx]

Supplementary Table 3: Sociodemographic characteristics of the Autistic, Autistic without intellectual disability, and Autistic with intellectual disability youth populations and their respective 1:10 propensity score matched non-Autistic comparison populations.

|  | Autistic | non-Autistic | Autistic without intellectual disability | non-Autistic | Autistic with intellectual disability | non-Autistic |
| --- | --- | --- | --- | --- | --- | --- |
|  | n (%) | n (%) | n (%) | n (%) | n (%) | n (%) |
| Total | 19,149 | 191,496 | 13,578 | 135,780 | 5,571 | 55,710 |
| *Sex* |  |  |  |  |  |  |
| Female | 4140 (21.6) | 41409 (21.6) | 2862 (21.1) | 28629 (21.1) | 1278 (22.9) | 12783 (22.9) |
| Male | 15009 (78.4) | 150087 (78.4) | 10716 (78.9) | 107151 (78.9) | 4293 (77.1) | 42927 (77.1) |
| *Age (years)* |  |  |  |  |  |  |
| 0-4 | 2034 (10.6) | 20367 (10.6) | 1362 (10) | 13629 (10) | 672 (12.1) | 6730 (12.1) |
| 5-9 | 4917 (25.7) | 49155 (25.7) | 3453 (25.4) | 34539 (25.4) | 1464 (26.3) | 14635 (26.3) |
| 10-14 | 5091 (26.6) | 50928 (26.6) | 3717 (27.4) | 37191 (27.4) | 1374 (24.7) | 13728 (24.6) |
| 15-19 | 4143 (21.6) | 41412 (21.6) | 2958 (21.8) | 29583 (21.8) | 1185 (21.3) | 11822 (21.2) |
| 20-24 | 2964 (15.5) | 29619 (15.5) | 2085 (15.4) | 20841 (15.3) | 879 (15.8) | 8785 (15.8) |
| *Ethnicity* |  |  |  |  |  |  |
| Māori | 4620 (24.1) | 46215 (24.1) | 3156 (23.2) | 31551 (23.2) | 1464 (26.3) | 14665 (26.3) |
| Pacific | 1923 (10) | 19209 (10) | 1020 (7.5) | 10182 (7.5) | 903 (16.2) | 9035 (16.2) |
| Non-Māori/non-Pacific | 13323 (69.6) | 133230 (69.6) | 9828 (72.4) | 98268 (72.4) | 3495 (62.7) | 34965 (62.8) |
| *Deprivation quintile* |  |  |  |  |  |  |
| 1 (least deprived) | 3675 (19.2) | 36765 (19.2) | 2724 (20.1) | 27234 (20.1) | 951 (17.1) | 9525 (17.1) |
| 2 | 3639 (19) | 36393 (19) | 2631 (19.4) | 26328 (19.4) | 1008 (18.1) | 10070 (18.1) |
| 3 | 3714 (19.4) | 37125 (19.4) | 2754 (20.3) | 27537 (20.3) | 960 (17.2) | 9585 (17.2) |
| 4 | 3894 (20.3) | 38931 (20.3) | 2796 (20.6) | 27984 (20.6) | 1098 (19.7) | 10960 (19.7) |
| 5 (most deprived) | 4224 (22.1) | 42270 (22.1) | 2670 (19.7) | 26700 (19.7) | 1554 (27.9) | 15560 (27.9) |
| *Urban/Rural* |  |  |  |  |  |  |
| urban | 17067 (89.1) | 170691 (89.1) | 12003 (88.4) | 120030 (88.4) | 5064 (90.9) | 50650 (90.9) |
| rural | 2079 (10.9) | 20796 (10.9) | 1575 (11.6) | 15750 (11.6) | 504 (9.1) | 5050 (9.1) |
