# Supplementary material for: Health service utilization among autistic youth in Aotearoa New Zealand: A nationwide cross-sectional study
Source: Autism. 2024 Dec 3;29(5):1143–56. doi: 10.1177/13623613241298352 (PMC12038068; doi:10.1177/13623613241298352)
Supplement: sj-docx-4-aut-10.1177_13623613241298352 – Supplemental material for Health service utilization among autistic youth in Aotearoa New Zealand: A nationwide cross-sectional study [file sj-docx-4-aut-10.1177_13623613241298352.docx]

Supplementary Table 4: Summary table of crude observed HSU outcomes by autism status, 2019

|  | Autism |  | non-Autism |  | Autism w/o ID | | Autism w/ ID | |
| --- | --- | --- | --- | --- | --- | --- | --- | --- |
|  | n | % | n | % | n | % | n | % |
| Hospitalization | |  |  |  |  |  |  |  |
| Yes | 2,427 | 12.5 | 161,217 | 10.3 | 1,485 | 10.7 | 942 | 16.6 |
| No | 17,052 | 87.5 | 1,400,061 | 89.7 | 12,336 | 89.3 | 4,716 | 83.4 |
| PAH |  |  |  |  |  |  |  |  |
| Yes | 1,827 | 9.4 | 83,136 | 5.3 | 1,146 | 8.3 | 681 | 12.0 |
| No | 17,652 | 90.6 | 1,478,142 | 94.7 | 12,675 | 91.7 | 4,977 | 88.0 |
| Self-harm |  |  |  |  |  |  |  |  |
| Yes | 129 | 0.7 | 3,621 | 0.2 | 108 | 0.8 | 21 | 0.4 |
| No | 19,350 | 99.3 | 1,557,657 | 99.8 | 13,713 | 99.2 | 5,637 | 99.6 |
| Emergency Department | |  |  |  |  |  |  |  |
| Yes | 3,372 | 17.3 | 253,767 | 16.3 | 2,346 | 17.0 | 1,026 | 18.1 |
| No | 16,107 | 82.7 | 1,307,511 | 83.7 | 11,475 | 83.0 | 4,632 | 81.9 |
| Outpatient |  |  |  |  |  |  |  |  |
| Yes | 10,131 | 52.0 | 387,609 | 24.8 | 6,822 | 49.4 | 3,309 | 58.5 |
| No | 9,348 | 48.0 | 1,173,669 | 75.2 | 6,999 | 50.6 | 2,349 | 41.5 |
| Psychiatric inpatient | |  |  |  |  |  |  |  |
| Yes | 210 | 1.1 | 3,006 | 0.2 | 153 | 1.1 | 57 | 1.0 |
| No | 19,269 | 98.9 | 1,558,272 | 99.8 | 13,668 | 98.9 | 5,601 | 99.0 |
| Psychiatric outpatient | |  |  |  |  |  |  |  |
| Yes | 2,916 | 15.0 | 42,060 | 2.7 | 2,241 | 16.2 | 675 | 11.9 |
| No | 16,563 | 85.0 | 1,519,218 | 97.3 | 11,580 | 83.8 | 4,983 | 88.1 |
| Psychotropic pharmaceutical dispensing | | |  |  |  |  |  |  |
| Yes | 6,942 | 35.6 | 79,398 | 5.1 | 4,842 | 35.0 | 2,100 | 37.1 |
| No | 12,537 | 64.4 | 1,481,880 | 94.9 | 8,979 | 65.0 | 3,558 | 62.9 |
| Non-psychotropic pharmaceutical dispensing | | | |  |  |  |  |  |
| Yes | 14,268 | 73.2 | 1,057,938 | 67.8 | 9,900 | 71.6 | 4,368 | 77.2 |
| No | 5,211 | 26.8 | 503,340 | 32.2 | 3,921 | 28.4 | 1,290 | 22.8 |
| Primary Health Care enrolment | | |  |  |  |  |  |  |
| Yes | 19,290 | 99.0 | 1,385,607 | 88.7 | 13,683 | 99.0 | 5,607 | 99.1 |
| No | 189 | 1.0 | 175,671 | 11.3 | 138 | 1.0 | 51 | 0.9 |
| Accident compensation scheme | | |  |  |  |  |  |  |
| Yes | 4,611 | 23.7 | 441,132 | 28.3 | 3,348 | 24.2 | 1,263 | 22.3 |
| No | 14,868 | 76.3 | 1,120,146 | 71.7 | 10,473 | 75.8 | 4,395 | 77.7 |
| Notes: PAH = respiratory conditions, dental conditions, gastrointestinal diseases, nutrition deficiency and anaemia, cardiovascular diseases, otitis media, dermatological conditions, diabetes complications, kidney, urinary tract infection, sexually transmitted infections, vaccine-preventable diseases, meningococcal infection, epilepsy, other non-injury conditions, unintentional injuries, intentional injuries. | | | | | | | | |
