# Supplementary material for: Health service utilization among autistic youth in Aotearoa New Zealand: A nationwide cross-sectional study
Source: Autism. 2024 Dec 3;29(5):1143–56. doi: 10.1177/13623613241298352 (PMC12038068; doi:10.1177/13623613241298352)
Supplement: sj-docx-5-aut-10.1177_13623613241298352 – Supplemental material for Health service utilization among autistic youth in Aotearoa New Zealand: A nationwide cross-sectional study [file sj-docx-5-aut-10.1177_13623613241298352.docx]

Table 6: Any inpatient hospitalization by sociodemographic sub-group and autism status

|  | Autism | | non-autism | | Autism without ID | | Autism with ID | |
| --- | --- | --- | --- | --- | --- | --- | --- | --- |
|  | n | % | n | % | n | % | n | % |
| Total | 2,427 | 12.5 | 161,217 | 10.3 | 1,485 | 10.7 | 942 | 16.6 |
| *Sex* |  |  |  |  |  |  |  |  |
| Male | 1,788 | 11.7 | 78,207 | 9.8 | 1,104 | 10.1 | 684 | 15.7 |
| Female | 639 | 15.1 | 83,010 | 10.9 | 381 | 13.0 | 258 | 19.9 |
| *Age (years)* |  |  |  |  |  |  |  |  |
| 0-4 | 447 | 21.7 | 81,627 | 27.9 | 291 | 21.1 | 156 | 22.9 |
| 5-9 | 771 | 15.4 | 20,766 | 6.6 | 483 | 13.7 | 288 | 19.4 |
| 10-14 | 501 | 9.7 | 13,701 | 4.3 | 321 | 8.5 | 180 | 12.9 |
| 15-19 | 423 | 10.0 | 17,532 | 5.7 | 234 | 7.8 | 189 | 15.8 |
| 20-24 | 288 | 9.5 | 27,594 | 8.4 | 159 | 7.5 | 129 | 14.4 |
| *Ethnicity* |  |  |  |  |  |  |  |  |
| Māori | 717 | 15.3 | 50,973 | 12.5 | 447 | 13.9 | 270 | 18.1 |
| Pacific | 315 | 16.2 | 25,893 | 12.1 | 141 | 13.7 | 174 | 19.1 |
| non-Māori/non-Pacific | 1,521 | 10.9 | 92,763 | 8.9 | 954 | 9.3 | 567 | 15.2 |
| *Area Level Deprivation (New Zealand Deprivation Index)* | | | | |  |  |  |  |
| 1 (least deprived) | 381 | 10.4 | 27,114 | 8.7 | 243 | 8.9 | 138 | 14.5 |
| 2 | 417 | 11.5 | 27,711 | 9.6 | 249 | 9.5 | 168 | 16.7 |
| 3 | 426 | 11.5 | 29,073 | 10.2 | 276 | 10.0 | 150 | 15.7 |
| 4 | 543 | 13.9 | 32,289 | 11.0 | 342 | 12.2 | 201 | 18.4 |
| 5 (most deprived) | 633 | 15.0 | 42,258 | 12.1 | 357 | 13.4 | 276 | 17.7 |
| missing | 27 | 8.2 | 2,772 | 9.3 | 18 | 7.4 | 9 | 10.3 |
| Urban/Rural residence | |  |  |  |  |  |  |  |
| Urban | 2,151 | 12.6 | 137,997 | 10.3 | 1,299 | 10.8 | 852 | 16.8 |
| Rural | 246 | 11.8 | 20,853 | 10.6 | 165 | 10.5 | 81 | 16.1 |
| missing | 30 | 10.1 | 2,367 | 9.1 | 21 | 9.9 | 9 | 10.7 |

Table 7: Any PAH hospitalization by sociodemographic sub-group and autism status

|  | Autism | | non-autism | | Autism without ID | | Autism with ID | |
| --- | --- | --- | --- | --- | --- | --- | --- | --- |
|  | n | % | n | % | n | % | n | % |
| Total | 1,827 | 9.4 | 83,136 | 5.3 | 1,146 | 8.3 | 681 | 12.0 |
| *Sex* |  |  |  |  |  |  |  |  |
| Male | 1,365 | 8.9 | 44,298 | 5.6 | 855 | 7.8 | 510 | 11.7 |
| Female | 462 | 10.9 | 38,838 | 5.1 | 291 | 9.9 | 171 | 13.2 |
| *Age (years)* |  |  |  |  |  |  |  |  |
| 0-4 | 339 | 16.4 | 30,642 | 10.5 | 225 | 16.3 | 114 | 16.7 |
| 5-9 | 624 | 12.5 | 17,475 | 5.6 | 390 | 11.1 | 234 | 15.8 |
| 10-14 | 330 | 6.4 | 9,285 | 2.9 | 204 | 5.4 | 126 | 9.0 |
| 15-19 | 312 | 7.4 | 11,727 | 3.8 | 186 | 6.2 | 126 | 10.5 |
| 20-24 | 225 | 7.4 | 14,004 | 4.2 | 141 | 6.6 | 84 | 9.4 |
| *Ethnicity* |  |  |  |  |  |  |  |  |
| Māori | 549 | 11.7 | 28,482 | 7.0 | 342 | 10.7 | 207 | 13.9 |
| Pacific | 252 | 13.0 | 15,927 | 7.4 | 114 | 11.1 | 138 | 15.1 |
| non-Māori/non-Pacific | 1,125 | 8.0 | 43,626 | 4.2 | 744 | 7.2 | 381 | 10.2 |
| *Area Level Deprivation (New Zealand Deprivation Index)* | | | | |  |  |  |  |
| 1 (least deprived) | 261 | 7.1 | 12,930 | 4.1 | 174 | 6.4 | 87 | 9.1 |
| 2 | 303 | 8.3 | 13,362 | 4.6 | 180 | 6.8 | 123 | 12.2 |
| 3 | 318 | 8.6 | 14,586 | 5.1 | 216 | 7.8 | 102 | 10.7 |
| 4 | 408 | 10.5 | 16,863 | 5.7 | 261 | 9.3 | 147 | 13.4 |
| 5 (most deprived) | 516 | 12.2 | 24,039 | 6.9 | 297 | 11.1 | 219 | 14.1 |
| missing | 21 | 6.4 | 1,356 | 4.5 | 18 | 7.4 | 3 | 3.4 |
| Urban/Rural residence | |  |  |  |  |  |  |  |
| Urban | 1,641 | 9.6 | 71,823 | 5.4 | 1,011 | 8.4 | 630 | 12.4 |
| Rural | 162 | 7.8 | 10,146 | 5.2 | 114 | 7.2 | 48 | 9.5 |
| missing | 24 | 8.1 | 1,167 | 4.5 | 21 | 9.9 | 3 | 3.6 |
| Notes: PAH = respiratory conditions, dental conditions, gastrointestinal diseases, nutrition deficiency and anaemia, cardiovascular diseases, otitis media, dermatological conditions, diabetes complications, kidney, urinary tract infection, sexually transmitted infections, vaccine-preventable diseases, meningococcal infection, epilepsy, other non-injury conditions, unintentional injuries, intentional injuries. | | | | | | | | |

Table 8: Any self-harm hospitalization by sociodemographic sub-group and autism status

|  | Autism | | non-autism | | Autism without ID | | Autism with ID | |
| --- | --- | --- | --- | --- | --- | --- | --- | --- |
|  | n | % | n | % | n | % | n | % |
| Total | 129 | 0.7 | 3,621 | 0.2 | 108 | 0.8 | 21 | 0.4 |
| *Sex* |  |  |  |  |  |  |  |  |
| Male | 66 | 0.4 | 960 | 0.1 | 57 | 0.5 | 9 | 0.2 |
| Female | 63 | 1.5 | 2,661 | 0.3 | 51 | 1.7 | 12 | 0.9 |
| *Age (years)* |  |  |  |  |  |  |  |  |
| 0-4 | ..S | n/a | ..S | n/a | ..S | n/a | ..S | n/a |
| 5-9 | ..S | n/a | ..S | n/a | ..S | n/a | ..S | n/a |
| 10-14 | 9 | 0.2 | 273 | 0.1 | ..S | n/a | ..S | n/a |
| 15-19 | 75 | 1.8 | 1,788 | 0.6 | 69 | 2.3 | 6 | 0.5 |
| 20-24 | 48 | 1.6 | 1,560 | 0.5 | 36 | 1.7 | 12 | 1.3 |
| *Ethnicity* |  |  |  |  |  |  |  |  |
| Māori | 30 | 0.6 | 1,317 | 0.4 | 21 | 0.7 | 9 | 0.6 |
| Pacific | 12 | 0.6 | 432 | 0.1 | ..S | n/a | ..S | n/a |
| non-Māori/non-Pacific | 99 | 0.7 | 2,052 | 0.6 | 87 | 0.8 | 12 | 0.3 |
| *Area Level Deprivation (New Zealand Deprivation Index)* | | | | |  |  |  |  |
| 1 (least deprived) | 21 | 0.6 | 513 | 0.2 | ..S | n/a | ..S | n/a |
| 2 | 21 | 0.6 | 618 | 0.2 | ..S | n/a | ..S | n/a |
| 3 | 24 | 0.6 | 651 | 0.2 | ..S | n/a | ..S | n/a |
| 4 | 36 | 0.9 | 822 | 0.3 | ..S | n/a | ..S | n/a |
| 5 (most deprived) | 27 | 0.6 | 969 | 0.3 | ..S | n/a | ..S | n/a |
| missing | 0 | 0.0 | 48 | 0.0 | ..S | n/a | ..S | n/a |
| Urban/Rural residence | |  |  |  |  |  |  |  |
| Urban | 120 | 0.7 | 3,258 | 1.0 | ..S | n/a | ..S | n/a |
| Rural | 9 | 0.4 | 324 | 0.1 | ..S | n/a | ..S | n/a |
| missing | 0 | 0.0 | 39 | 0.0 | ..S | n/a | ..S | n/a |

Table 9: Any Emergency Department presentation by sociodemographic sub-group and autism status

|  | Autism | | non-autism | | Autism w/o ID | | Autism w/ ID | |
| --- | --- | --- | --- | --- | --- | --- | --- | --- |
|  | n | % | n | % | n | % | n | % |
| Total | 3,372 | 17.3 | 253,767 | 16.3 | 2,346 | 17.0 | 1,026 | 18.1 |
| *Sex* |  |  |  |  |  |  |  |  |
| Male | 2,493 | 16.3 | 132,402 | 16.6 | 1,746 | 16.0 | 747 | 17.1 |
| Female | 879 | 20.8 | 121,365 | 15.9 | 600 | 20.5 | 279 | 21.5 |
| *Age (years)* |  |  |  |  |  |  |  |  |
| 0-4 | 651 | 31.6 | 73,035 | 24.9 | 447 | 32.4 | 204 | 30.0 |
| 5-9 | 849 | 17.0 | 41,175 | 13.1 | 591 | 16.8 | 258 | 17.4 |
| 10-14 | 648 | 12.5 | 36,630 | 11.5 | 471 | 12.5 | 177 | 12.7 |
| 15-19 | 654 | 15.5 | 45,963 | 15.0 | 447 | 14.8 | 207 | 17.3 |
| 20-24 | 561 | 18.5 | 56,964 | 17.3 | 387 | 18.1 | 174 | 19.5 |
| *Ethnicity* |  |  |  |  |  |  |  |  |
| Māori | 915 | 19.5 | 82,008 | 20.2 | 624 | 19.4 | 291 | 19.5 |
| Pacific | 396 | 20.4 | 40,887 | 19.1 | 195 | 19.0 | 201 | 22.0 |
| non-Māori/non-Pacific | 2,202 | 15.7 | 143,232 | 13.8 | 1,608 | 15.6 | 594 | 15.9 |
| *Area Level Deprivation (New Zealand Deprivation Index)* | | | | |  |  |  |  |
| 1 (least deprived) | 525 | 14.3 | 40,605 | 13.0 | 390 | 14.3 | 135 | 14.2 |
| 2 | 576 | 15.8 | 41,895 | 14.5 | 405 | 15.4 | 171 | 17.0 |
| 3 | 618 | 16.7 | 45,444 | 15.9 | 459 | 16.7 | 159 | 16.6 |
| 4 | 747 | 19.2 | 52,428 | 17.9 | 531 | 19.0 | 216 | 19.7 |
| 5 (most deprived) | 849 | 20.1 | 69,045 | 19.8 | 528 | 19.8 | 321 | 20.6 |
| missing | 57 | 17.3 | 4,350 | 14.5 | 33 | 13.6 | 24 | 27.6 |
| Urban/Rural residence | |  |  |  |  |  |  |  |
| Urban | 2,976 | 17.4 | 218,151 | 16.3 | 2,049 | 17.0 | 927 | 18.3 |
| Rural | 342 | 16.5 | 31,860 | 16.2 | 264 | 16.8 | 78 | 15.5 |
| missing | 54 | 18.2 | 3,756 | 14.4 | 33 | 15.5 | 21 | 25.0 |

Table 10: Any outpatient specialty visit by sociodemographic sub-group and autism status

|  | Autism | | non-autism | | Autism w/o ID | | Autism w/ ID | |
| --- | --- | --- | --- | --- | --- | --- | --- | --- |
|  | n | % | n | % | n | % | n | % |
| Total | 10,131 | 52.0 | 387,609 | 24.8 | 6,822 | 49.4 | 3,309 | 58.5 |
| *Sex* |  |  |  |  |  |  |  |  |
| Male | 7,797 | 51.1 | 196,323 | 24.6 | 5,283 | 48.5 | 2,514 | 57.7 |
| Female | 2,334 | 55.3 | 191,286 | 25.0 | 1,539 | 52.6 | 795 | 61.2 |
| *Age (years)* |  |  |  |  |  |  |  |  |
| 0-4 | 1,461 | 70.9 | 97,041 | 33.1 | 990 | 71.7 | 471 | 69.2 |
| 5-9 | 3,594 | 71.9 | 112,848 | 35.9 | 2,523 | 71.8 | 1,071 | 72.1 |
| 10-14 | 2,898 | 56.0 | 91,257 | 28.7 | 2,043 | 54.1 | 855 | 61.3 |
| 15-19 | 1,410 | 33.4 | 40,209 | 13.1 | 846 | 28.0 | 564 | 47.0 |
| 20-24 | 765 | 25.3 | 46,248 | 14.0 | 417 | 19.5 | 348 | 38.9 |
| *Ethnicity* |  |  |  |  |  |  |  |  |
| Māori | 2,745 | 58.4 | 109,638 | 27.0 | 1,836 | 57.2 | 909 | 61.0 |
| Pacific | 1,122 | 57.8 | 44,202 | 20.6 | 570 | 55.4 | 552 | 60.5 |
| non-Māori/non-Pacific | 6,696 | 47.8 | 248,478 | 23.9 | 4,665 | 45.4 | 2,031 | 54.5 |
| *Area Level Deprivation (New Zealand Deprivation Index)* | | | | |  |  |  |  |
| 1 (least deprived) | 1,719 | 46.7 | 80,148 | 25.6 | 1,212 | 44.5 | 507 | 53.1 |
| 2 | 1,785 | 49.1 | 72,726 | 25.1 | 1,206 | 45.8 | 579 | 57.4 |
| 3 | 1,911 | 51.5 | 71,367 | 25.0 | 1,344 | 48.8 | 567 | 59.2 |
| 4 | 2,160 | 55.5 | 73,803 | 25.1 | 1,479 | 52.8 | 681 | 62.2 |
| 5 (most deprived) | 2,394 | 56.6 | 82,146 | 23.5 | 1,461 | 54.7 | 933 | 59.9 |
| missing | 162 | 49.1 | 7,419 | 24.8 | 120 | 49.4 | 42 | 48.3 |
| Urban/Rural residence | |  |  |  |  |  |  |  |
| Urban | 8,865 | 51.8 | 319,845 | 23.9 | 5,901 | 49.0 | 2,964 | 58.5 |
| Rural | 1,119 | 53.8 | 61,404 | 31.3 | 816 | 51.8 | 303 | 60.1 |
| missing | 147 | 49.5 | 6,360 | 24.4 | 105 | 49.3 | 42 | 50.0 |

Table 11: Any psychiatric inpatient hospitalization by sociodemographic sub-group and autism status

|  | Autism | | non-autism | | Autism w/o ID | | Autism w/ ID | |
| --- | --- | --- | --- | --- | --- | --- | --- | --- |
|  | n | % | n | % | n | % | n | % |
| Total | 210 | 1.1 | 3,006 | 0.2 | 153 | 1.1 | 57 | 1.0 |
| *Sex* |  |  |  |  |  |  |  |  |
| Male | 120 | 0.8 | 1,401 | 0.2 | 81 | 0.7 | 39 | 0.9 |
| Female | 90 | 2.1 | 1,605 | 0.2 | 72 | 2.5 | 18 | 1.4 |
| *Age (years)* |  |  |  |  |  |  |  |  |
| 0-4 | ..S | n/a | ..S | n/a | ..S | n/a | ..S | n/a |
| 5-9 | ..S | n/a | ..S | n/a | ..S | n/a | ..S | n/a |
| 10-14 | 27 | 0.5 | 120 | 0.0 | 21 | 0.6 | 6 | 0.4 |
| 15-19 | 93 | 2.2 | 1,209 | 0.4 | 72 | 2.4 | 21 | 1.8 |
| 20-24 | 87 | 2.9 | 1,617 | 0.5 | 57 | 2.7 | 30 | 3.4 |
| *Ethnicity* |  |  |  |  |  |  |  |  |
| Māori | 57 | 1.2 | 1,254 | 0.3 | 33 | 1.0 | 24 | 1.6 |
| Pacific | ..S | n/a | 333 | 0.2 | ..S | n/a | ..S | n/a |
| non-Māori/non-Pacific | 150 | 1.1 | 1,569 | 0.2 | 117 | 1.1 | 33 | 0.9 |
| *Area Level Deprivation (New Zealand Deprivation Index)* | | | | |  |  |  |  |
| 1 (least deprived) | 30 | 0.8 | 402 | 0.1 | 21 | 0.8 | 9 | 0.9 |
| 2 | 42 | 1.2 | 396 | 0.1 | 30 | 1.1 | 12 | 1.2 |
| 3 | 39 | 1.1 | 525 | 0.2 | 27 | 1.0 | 12 | 1.3 |
| 4 | 51 | 1.3 | 705 | 0.2 | 36 | 1.3 | 15 | 1.4 |
| 5 (most deprived) | 48 | 1.1 | 921 | 0.3 | 36 | 1.3 | 12 | 0.8 |
| missing | 0 | 0.0 | 57 | 0.2 | 3 | 1.2 | -3 | -3.4 |
| Urban/Rural residence | |  |  |  |  |  |  |  |
| Urban | 189 | 1.1 | 2,652 | 0.2 | 135 | 1.1 | 54 | 1.1 |
| Rural | 21 | 1.0 | 303 | 0.2 | ..S | n/a | ..S | n/a |
| missing | 0 | 0.0 | 51 | 0.2 | ..S | n/a | ..S | n/a |

Table 12: Any mental health outpatient specialty visit by sociodemographic sub-group and autism status

|  | Autism | | non-autism | | Autism w/o ID | | Autism w/ ID | |
| --- | --- | --- | --- | --- | --- | --- | --- | --- |
|  | n | % | n | % | n | % | n | % |
| Total | 2,916 | 15.0 | 42,060 | 2.7 | 2,241 | 16.2 | 675 | 11.9 |
| *Sex* |  |  |  |  |  |  |  |  |
| Male | 2,091 | 13.7 | 20,595 | 2.6 | 1,587 | 14.6 | 504 | 11.6 |
| Female | 825 | 19.5 | 21,465 | 2.8 | 654 | 22.4 | 171 | 13.2 |
| *Age (years)* |  |  |  |  |  |  |  |  |
| 0-4 | 42 | 2.0 | 696 | 0.2 | 30 | 2.2 | 12 | 1.8 |
| 5-9 | 477 | 9.5 | 3,954 | 1.3 | 399 | 11.4 | 78 | 5.3 |
| 10-14 | 948 | 18.3 | 8,541 | 2.7 | 783 | 20.7 | 165 | 11.8 |
| 15-19 | 930 | 22.0 | 15,870 | 5.2 | 705 | 23.4 | 225 | 18.8 |
| 20-24 | 510 | 16.8 | 13,002 | 3.9 | 321 | 15.0 | 189 | 21.1 |
| *Ethnicity* |  |  |  |  |  |  |  |  |
| Māori | 699 | 14.9 | 14,313 | 3.5 | 498 | 15.5 | 201 | 13.5 |
| Pacific | 156 | 8.0 | 4,923 | 2.3 | 99 | 9.6 | 57 | 6.3 |
| non-Māori/non-Pacific | 2,133 | 15.2 | 24,738 | 2.4 | 1,689 | 16.4 | 444 | 11.9 |
| *Area Level Deprivation (New Zealand Deprivation Index)* | | | | |  |  |  |  |
| 1 (least deprived) | 510 | 13.9 | 6,507 | 2.1 | 408 | 15.0 | 102 | 10.7 |
| 2 | 546 | 15.0 | 6,846 | 2.4 | 417 | 15.8 | 129 | 12.8 |
| 3 | 576 | 15.5 | 7,722 | 2.7 | 447 | 16.2 | 129 | 13.5 |
| 4 | 633 | 16.3 | 9,096 | 3.1 | 495 | 17.7 | 138 | 12.6 |
| 5 (most deprived) | 591 | 14.0 | 11,319 | 3.2 | 423 | 15.8 | 168 | 10.8 |
| missing | 60 | 18.2 | 570 | 1.9 | 51 | 21.0 | 9 | 10.3 |
| Urban/Rural residence | |  |  |  |  |  |  |  |
| Urban | 2,502 | 14.6 | 37,074 | 2.8 | 1,917 | 15.9 | 585 | 11.5 |
| Rural | 351 | 16.9 | 4,503 | 2.3 | 276 | 17.5 | 75 | 14.9 |
| missing | 63 | 21.2 | 483 | 1.9 | 48 | 22.5 | 15 | 17.9 |

Table 13: Any psychotropic medication dispensing by sociodemographic sub-group and autism status

|  | Autism | | non-autism | | Autism w/o ID | | Autism w/ ID | |
| --- | --- | --- | --- | --- | --- | --- | --- | --- |
|  | n | % | n | % | n | % | n | % |
| Total | 6,942 | 35.6 | 79,398 | 5.1 | 4,842 | 35.0 | 2,100 | 37.1 |
| *Sex* |  |  |  |  |  |  |  |  |
| Male | 5,238 | 34.3 | 34,086 | 4.3 | 3,660 | 33.6 | 1,578 | 36.2 |
| Female | 1,704 | 40.3 | 45,312 | 5.9 | 1,182 | 40.4 | 522 | 40.2 |
| *Age (years)* |  |  |  |  |  |  |  |  |
| 0-4 | 180 | 8.7 | 342 | 0.1 | 114 | 8.3 | 66 | 9.7 |
| 5-9 | 1,434 | 28.7 | 4,713 | 1.5 | 1,044 | 29.7 | 390 | 26.3 |
| 10-14 | 2,214 | 42.8 | 9,258 | 2.9 | 1,620 | 42.9 | 594 | 42.6 |
| 15-19 | 1,842 | 43.7 | 23,382 | 7.6 | 1,269 | 42.0 | 573 | 47.8 |
| 20-24 | 1,269 | 41.9 | 41,700 | 12.7 | 795 | 37.3 | 474 | 53.0 |
| *Ethnicity* |  |  |  |  |  |  |  |  |
| Māori | 1,500 | 31.9 | 18,552 | 4.6 | 990 | 30.8 | 510 | 34.2 |
| Pacific | 423 | 21.8 | 4,524 | 2.1 | 219 | 21.3 | 204 | 22.4 |
| non-Māori/non-Pacific | 5,205 | 37.2 | 58,131 | 5.6 | 3,738 | 36.4 | 1,467 | 39.3 |
| *Area Level Deprivation (New Zealand Deprivation Index)* | | | | |  |  |  |  |
| 1 (least deprived) | 1,392 | 37.8 | 16,203 | 5.2 | 996 | 36.6 | 396 | 41.5 |
| 2 | 1,335 | 36.7 | 15,180 | 5.2 | 924 | 35.1 | 411 | 40.8 |
| 3 | 1,356 | 36.5 | 15,576 | 5.5 | 975 | 35.4 | 381 | 39.8 |
| 4 | 1,413 | 36.3 | 16,224 | 5.5 | 1,005 | 35.9 | 408 | 37.3 |
| 5 (most deprived) | 1,314 | 31.1 | 14,949 | 4.3 | 852 | 31.9 | 462 | 29.7 |
| missing | 132 | 40.0 | 1,266 | 4.2 | 90 | 37.0 | 42 | 48.3 |
| Urban/Rural residence | |  |  |  |  |  |  |  |
| Urban | 6,084 | 35.6 | 70,092 | 5.2 | 4,215 | 35.0 | 1,869 | 36.9 |
| Rural | 738 | 35.5 | 8,259 | 4.2 | 546 | 34.7 | 192 | 38.1 |
| missing | 120 | 40.4 | 1,047 | 4.0 | 81 | 38.0 | 39 | 46.4 |

Table 14: Any non-psychotropic medication dispensing by sociodemographic sub-group and autism status

|  | Autism | | non-autism | | Autism w/o ID | | Autism w/ ID | |
| --- | --- | --- | --- | --- | --- | --- | --- | --- |
|  | n | % | n | % | n | % | n | % |
| Total | 14,268 | 73.2 | 1,057,938 | 67.8 | 9,900 | 71.6 | 4,368 | 77.2 |
| *Sex* |  |  |  |  |  |  |  |  |
| Male | 10,884 | 71.3 | 507,459 | 63.6 | 7,599 | 69.7 | 3,285 | 75.4 |
| Female | 3,384 | 80.1 | 550,479 | 72.1 | 2,301 | 78.7 | 1,083 | 83.4 |
| *Age (years)* |  |  |  |  |  |  |  |  |
| 0-4 | 1,869 | 90.7 | 245,196 | 83.7 | 1,242 | 90.0 | 627 | 92.1 |
| 5-9 | 3,966 | 79.4 | 228,330 | 72.6 | 2,757 | 78.5 | 1,209 | 81.4 |
| 10-14 | 3,582 | 69.3 | 194,949 | 61.4 | 2,568 | 68.0 | 1,014 | 72.7 |
| 15-19 | 2,811 | 66.6 | 187,569 | 61.2 | 1,956 | 64.8 | 855 | 71.3 |
| 20-24 | 2,043 | 67.5 | 201,891 | 61.2 | 1,380 | 64.7 | 663 | 74.2 |
| *Ethnicity* |  |  |  |  |  |  |  |  |
| Māori | 3,399 | 72.3 | 278,973 | 68.7 | 2,310 | 72.0 | 1,089 | 73.0 |
| Pacific | 1,530 | 78.8 | 153,753 | 71.7 | 792 | 77.0 | 738 | 80.9 |
| non-Māori/non-Pacific | 9,873 | 70.5 | 670,782 | 64.5 | 7,110 | 69.2 | 2,763 | 74.1 |
| *Area Level Deprivation (New Zealand Deprivation Index)* | | | | |  |  |  |  |
| 1 (least deprived) | 2,652 | 72.1 | 212,877 | 68.0 | 1,929 | 70.8 | 723 | 75.8 |
| 2 | 2,622 | 72.1 | 195,636 | 67.6 | 1,833 | 69.7 | 789 | 78.3 |
| 3 | 2,679 | 72.2 | 192,522 | 67.4 | 1,941 | 70.5 | 738 | 77.1 |
| 4 | 2,886 | 74.1 | 198,915 | 67.7 | 2,037 | 72.8 | 849 | 77.5 |
| 5 (most deprived) | 3,189 | 75.4 | 239,958 | 68.7 | 1,986 | 74.4 | 1,203 | 77.3 |
| missing | 240 | 72.7 | 18,030 | 60.2 | 174 | 71.6 | 66 | 75.9 |
| Urban/Rural residence | |  |  |  |  |  |  |  |
| Urban | 12,645 | 73.9 | 916,032 | 68.4 | 8,697 | 72.3 | 3,948 | 77.9 |
| Rural | 1,413 | 68.0 | 126,447 | 64.5 | 1,059 | 67.2 | 354 | 70.2 |
| missing | 210 | 70.7 | 15,459 | 59.4 | 144 | 67.6 | 66 | 78.6 |

Table 15: Primary Health Care enrolment by sociodemographic sub-group and autism status

|  | Autism | | non-autism | | Autism w/o ID | | Autism w/ ID | |
| --- | --- | --- | --- | --- | --- | --- | --- | --- |
|  | n | % | n | % | n | % | n | % |
| Total | 19,290 | 99.0 | 1,385,607 | 88.7 | 13,683 | 99.0 | 5,607 | 99.1 |
| *Sex* |  |  |  |  |  |  |  |  |
| Male | 15,102 | 99.0 | 704,808 | 88.4 | 10,785 | 99.0 | 4,317 | 99.0 |
| Female | 4,188 | 99.1 | 680,799 | 89.1 | 2,898 | 99.1 | 1,290 | 99.3 |
| *Age (years)* |  |  |  |  |  |  |  |  |
| 0-4 | 1,956 | 94.9 | 210,027 | 71.7 | 1,296 | 93.9 | 660 | 96.9 |
| 5-9 | 4,938 | 98.8 | 303,717 | 96.5 | 3,477 | 99.0 | 1,461 | 98.4 |
| 10-14 | 5,160 | 99.8 | 306,690 | 96.5 | 3,765 | 99.7 | 1,395 | 100.0 |
| 15-19 | 4,215 | 99.9 | 284,850 | 93.0 | 3,018 | 100.0 | 1,197 | 99.8 |
| 20-24 | 3,024 | 99.9 | 280,323 | 85.0 | 2,130 | 99.9 | 894 | 100.0 |
| *Ethnicity* |  |  |  |  |  |  |  |  |
| Māori | 4,656 | 99.0 | 379,656 | 93.5 | 3,180 | 99.1 | 1,476 | 99.0 |
| Pacific | 1,905 | 98.1 | 193,095 | 90.0 | 1,005 | 97.7 | 900 | 98.7 |
| non-Māori/non-Pacific | 13,443 | 96.0 | 871,326 | 83.8 | 9,918 | 96.5 | 3,525 | 94.5 |
| *Area Level Deprivation (New Zealand Deprivation Index)* | | | | |  |  |  |  |
| 1 (least deprived) | 3,645 | 99.1 | 282,720 | 90.3 | 2,703 | 99.2 | 942 | 98.7 |
| 2 | 3,603 | 99.0 | 257,184 | 88.8 | 2,610 | 99.2 | 993 | 98.5 |
| 3 | 3,681 | 99.2 | 252,360 | 88.4 | 2,727 | 99.0 | 954 | 99.7 |
| 4 | 3,861 | 99.2 | 258,513 | 88.0 | 2,769 | 98.9 | 1,092 | 99.7 |
| 5 (most deprived) | 4,173 | 98.7 | 311,124 | 89.0 | 2,634 | 98.7 | 1,539 | 98.8 |
| missing | 327 | 99.1 | 23,706 | 79.2 | 240 | 98.8 | 87 | 100.0 |
| Urban/Rural residence | |  |  |  |  |  |  |  |
| Urban | 16,929 | 99.0 | 1,184,466 | 88.5 | 11,907 | 99.0 | 5,022 | 99.1 |
| Rural | 2,070 | 99.6 | 180,585 | 92.0 | 1,566 | 99.4 | 504 | 100.0 |
| missing | 291 | 98.0 | 20,556 | 79.0 | 210 | 98.6 | 81 | 96.4 |

Table 16: Any accident compensation corporation claim by sociodemographic sub-group and autism status

|  | Autism | | non-autism | | Autism w/o ID |  | Autism w/ ID |  |
| --- | --- | --- | --- | --- | --- | --- | --- | --- |
|  | n | % | n | % | n | % | n | % |
| Total | 4,611 | 23.7 | 441,132 | 28.3 | 3,348 | 24.2 | 1,263 | 22.3 |
| *Sex* |  |  |  |  |  |  |  |  |
| Male | 3,516 | 23.0 | 244,344 | 30.6 | 2,559 | 23.5 | 957 | 22.0 |
| Female | 1,095 | 25.9 | 196,788 | 25.8 | 789 | 27.0 | 306 | 23.6 |
| *Age (years)* |  |  |  |  |  |  |  |  |
| 0-4 | 567 | 27.5 | 64,587 | 22.0 | 384 | 27.8 | 183 | 26.9 |
| 5-9 | 1,170 | 23.4 | 79,170 | 25.2 | 843 | 24.0 | 327 | 22.0 |
| 10-14 | 1,269 | 24.5 | 103,767 | 32.7 | 954 | 25.3 | 315 | 22.6 |
| 15-19 | 921 | 21.8 | 97,086 | 31.7 | 669 | 22.2 | 252 | 21.0 |
| 20-24 | 678 | 22.4 | 96,519 | 29.3 | 498 | 23.3 | 180 | 20.1 |
| *Ethnicity* |  |  |  |  |  |  |  |  |
| Māori | 1,059 | 22.5 | 111,294 | 27.4 | 747 | 23.3 | 312 | 20.9 |
| Pacific | 426 | 21.9 | 57,138 | 26.6 | 231 | 22.4 | 195 | 21.4 |
| non-Māori/non-Pacific | 3,285 | 23.5 | 289,242 | 27.8 | 2,463 | 24.0 | 822 | 22.0 |
| *Area Level Deprivation (New Zealand Deprivation Index)* | | | | |  |  |  |  |
| 1 (least deprived) | 924 | 25.1 | 96,570 | 30.9 | 711 | 26.1 | 213 | 22.3 |
| 2 | 861 | 23.7 | 85,581 | 29.6 | 633 | 24.1 | 228 | 22.6 |
| 3 | 885 | 23.8 | 81,390 | 28.5 | 657 | 23.9 | 228 | 23.8 |
| 4 | 894 | 23.0 | 79,584 | 27.1 | 663 | 23.7 | 231 | 21.1 |
| 5 (most deprived) | 957 | 22.6 | 89,466 | 25.6 | 618 | 23.1 | 339 | 21.8 |
| missing | 90 | 27.3 | 8,541 | 28.5 | 66 | 27.2 | 24 | 27.6 |
| Urban/Rural residence | |  |  |  |  |  |  |  |
| Urban | 4,029 | 23.6 | 375,396 | 28.0 | 2,904 | 24.1 | 1,125 | 22.2 |
| Rural | 498 | 24.0 | 58,218 | 29.7 | 384 | 24.4 | 114 | 22.6 |
| missing | 84 | 28.3 | 7,518 | 28.9 | 60 | 28.2 | 24 | 28.6 |
